# Dosimetric impact of daily skin surface variations in kidney stereotactic ablative proton therapy

**DOI:** 10.1016/j.tipsro.2026.100428

**Published:** 2026-08-17

**Authors:** Yohan A. Walter, Olivia G. Moncrief, Chiachien J. Wang, James C. Henry, Mitchell Wolden, Emily G. Warren, Haley Clark, Amber Speir, Daniel B. Speir, Kelsi Hoffnung, Bethany Broekhoven, Philip F. Durham, Kaylee Kallam, Hsinshun T. Wu

**Affiliations:** a Department of Radiation Oncology, Willis Knighton Cancer Center, 2600 Kings Highway, Shreveport, Louisiana 71103, USA; b Clinical Research Department, University of Jamestown, 4190 26th Ave. S. Fargo, North Dakota 58104, USA; c School of Medicine, Louisiana State University Health Shreveport, 1501 Kings Highway, Shreveport, Louisiana 71103, USA

**Keywords:** stereotactic body radiation therapy, stereotactic radiotherapy, renal cell carcinoma, proton beam therapy, stereotactic body proton therapy, dose accumulation

## Abstract

**Purpose:**

Proton beam therapy (PBT) is a promising modality in kidney stereotactic ablative radiotherapy (SABR). However, the sensitivity of PBT to materials in the beam path enforces special considerations in patient positioning. In clinical practice, we observed significant variability in the skin surface, which may perturb delivered dose. Here, we characterize the impact of skin surface variations in kidney stereotactic body proton therapy (SBPT).

**Materials and methods:**

Patients treated with kidney SBPT between 2024 and 2025 were included. Assessment plans were created using 3.0 mm positioning uncertainty in robust optimization to deliver a prescription dose of 50 Gy(RBE) in 5 fractions to 99% of the target volume (iCTV). Virtual CTs were generated for dose accumulation by mapping HU from the planning scan onto the day-of-treatment CBCT. The assessment plan was mapped to each virtual CT set. The dose to 95–100% of the target (iCTV D95-Dmin) and the volume of healthy kidney receiving 10–50 Gy (V10-V50) were compared between planned and accumulated distributions.

**Results:**

Fifteen patients treated in 75 fractions were included. The depth of the tumor centroid along the beam path varied by up to 17.4 mm relative to the planned anatomy. Depth variations exceeded 5.0 mm in 22.7% of fractions, and resulted in lower iCTV D95, D98, D99, and Dmin (*p* < 0.01) compared to assessment plan distributions. The accumulated D99 dropped by up to 24.3%.

**Conclusion:**

Skin surface variations may compromise kidney SBPT treatment quality. Further investigation into mitigation strategies, such as adaptive radiotherapy, are merited.

## Highlights

• Daily skin surface inconsistency significantly perturbs delivered dose.
• Perturbations may compromise safety and efficacy.
• Adjustments to setup and optimization may mitigate perturbations.
• Online ART may be necessary in kidney SBPT.

## Introduction

Multiple recent studies have shown that, in both the metastatic [1], [2], [3], [4], [5] and primary tumor setting, [6], [7], [8], [9], [10], [11] stereotactic ablative radiotherapy (SABR) may effectively control renal cell carcinoma (RCC), the most common primary kidney tumor. [12], [13]

Considering the promising results in photon-based SABR, interest has grown in proton beam therapy (PBT)-based SABR for RCC. [14], [15], [16], [17] The physical characteristics of proton beams yield dosimetric effects which may be beneficial in the RCC setting, including higher biological effect within treated areas and reduced out-of-target dose compared to photon beams. [14], [15], [16], [18]

**Fig. 1:**
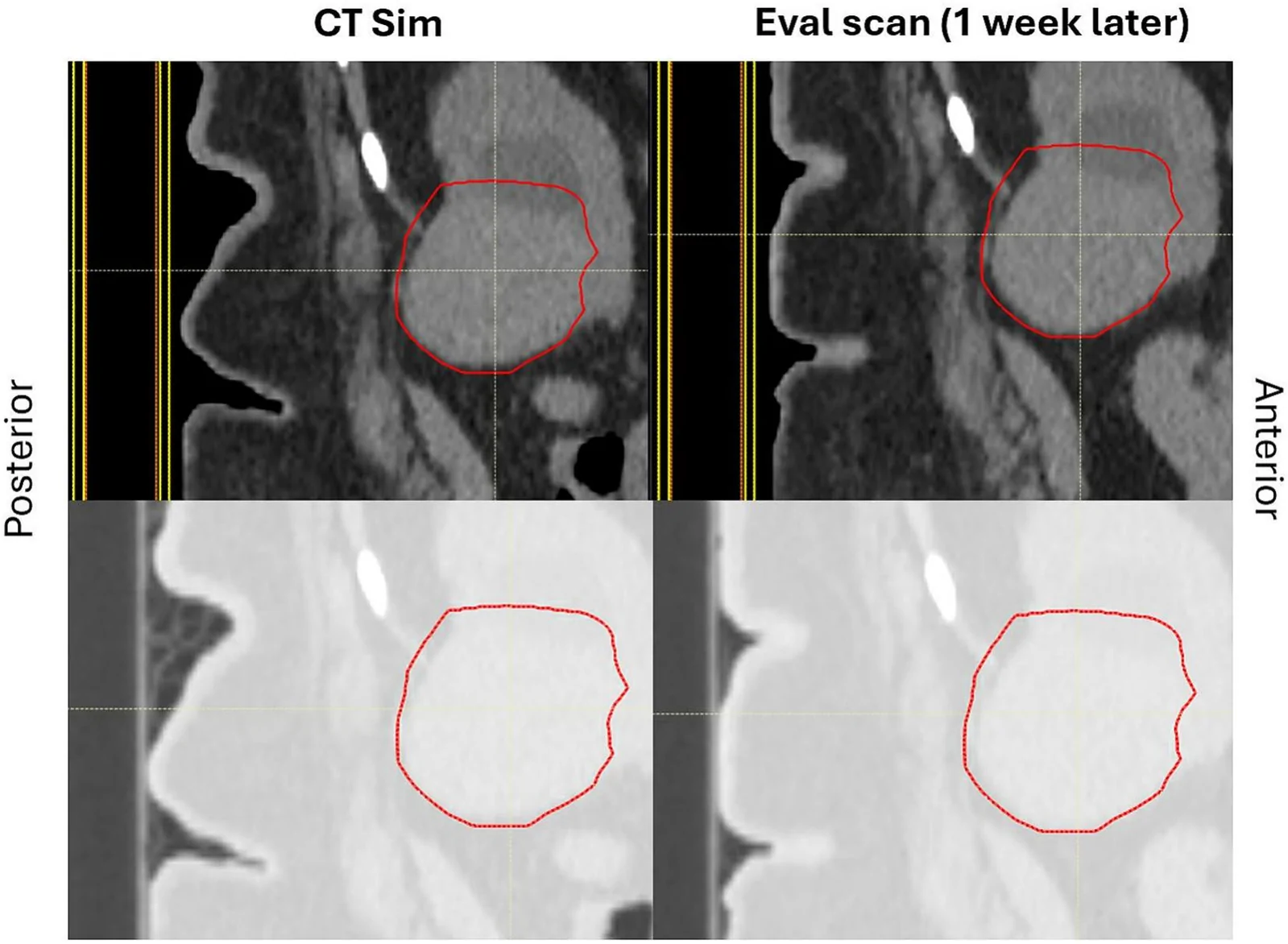
Comparison of posterior skin surface in initial CT simulation scan (left) versus an evaluation CT scan taken 1 week later (right). Viewing in the lung window (bottom row) reveals a sheet underneath the patient used for ease of positioning, creating large skin surface variability. The target (iCTV) is delineated in red. (For interpretation of the references to colour in this figure legend, the reader is referred to the web version of this article.)

Though PBT carries significant dosimetric advantages over x-ray-based therapy, [18] clinical protocols must feature additional quality control to ensure adequate consideration of PBT-specific beam delivery and patient positioning uncertainties. [19] In treatment of kidney tumors, the anatomical location poses several difficulties: first, kidney tumors are subject to respiration-induced motion. [20] Second, the absence of anatomical anchoring points, combined with the sensitivity of proton beams to materials in the beam path, limits immobilization options. [21] Finally, loose tissue in the abdomen can introduce daily variations in the radiological path length (Fig. 1).

Though respiratory motion and immobilization can be managed through various proton-friendly means, [22], [23] skin surface variations are often overlooked. Furthermore, standard online cone beam computed tomography (CBCT) protocols may not always include a sufficient field-of-view (FOV) to adequately assess skin surface positioning. Surface variations may thus present both safety and efficacy concerns in kidney SBPT; however, the impact on delivered dose has not yet been quantified in this setting.

The purpose of this work was to determine the impact of daily skin surface variations on delivered SBPT dose distributions. Using a density conversion and dose accumulation method, the delivered dose was modeled using daily treatment setup images. The results of this work may elucidate areas of concern in kidney SBPT. Additionally, results may offer insight into new mitigation strategies to account for daily skin surface variations.

## Materials and methods

### Patient cohort

Data were retrospectively collected for patients treated with 5-fraction SBPT for kidney tumors between 2024 and 2025 at the Willis Knighton Cancer Center (Shreveport, Louisiana, USA). Only patients with complete on-treatment imaging data were included. Demographic and tumor data, including target size, location relative to the renal hilum, and the patient's BMI, were recorded. The study received institutional review board approval (WCG IRB, 00000533). The requirement for informed consent was waived due to the retrospective nature of the study .

### CT simulation

Patients were simulated in the headfirst supine position. The immobilization setup used a wing board (Arm Shuttle, CQ Medical, Avondale, PA) with a vacuum bag or knee wedge under the patient's legs. No additional immobilization or positioning aids were placed near the abdomen or thorax.

Respiration-correlated computed tomography (4DCT) images were acquired on a Big Bore RT scanner (Philips, Andover, MA). Respiratory motion traces for 4D reconstruction were acquired using the Sentinel surface monitoring system (C-RAD AB, Stockholm, Sweden).

Following the initial simulation, patients were scheduled for an evaluation scan, in which the simulation process was repeated using the setup established in the initial session. Evaluation scans were acquired at 2–10 days following the initial simulation. Both the initial simulation and evaluation scans were acquired before initiating treatment.

Average intensity, maximum intensity (MIP), and ten phase-binned reconstructions were generated for both the initial and evaluation simulations and exported to the RayStation version 11A or 2023B (RaySearch Laboratories AB, Stockholm, Sweden) treatment planning system (TPS).

### Treatment planning

Organs at risk (OARs) were delineated on the average intensity simulation image set using AI-assisted auto-contouring (Limbus Contour v1.8, Limbus AI, Radformation, New York, NY). Physicians contoured the internal clinical target volume (iCTV) using the MIP. Structures were mapped to the evaluation scan via rigid registration and adjusted manually, as necessary.

To evaluate the impact of skin surface perturbations, two nonclinical treatment plans were generated for each patient case. Clinical plans were not used in any component of the current analysis. The assessment plans used a 2-field beam arrangement with posterior-anterior (P/A) and ipsilateral beams, to align with clinical planning strategies. Single-field uniform dose (SFUD) optimization with equal beam weighting was used to deliver the prescription dose of 5000 cGy (1.1 relative biological effect weighting, RBE) to 99.0% of the iCTV.

Robust optimization was performed with 3.0 mm positioning uncertainty, applied to the 99.0% target coverage and maximum dose planning objectives. Separately, an assessment plan which included the evaluation CT scan in the robust optimization was generated to determine if factoring both scans into dose planning sufficiently reduced sensitivity to beam perturbations.

As range uncertainty is intended to account for errors in stopping power conversion and proton interactions with matter, extra robustness introduced by the range uncertainty in optimization would potentially mask the effect of skin surface perturbations along the beam path. It was therefore excluded from optimization. The initial average intensity CT was used as the basis for dose calculation.

### Virtual CT generation and dose mapping

The large-FOV CBCTs taken during initial setup at each treated fraction were exported to the TPS. If at least one CBCT did not fully encompass the skin surface in the direction of incident beams, those cases were excluded from the analysis.

**Fig. 2:**
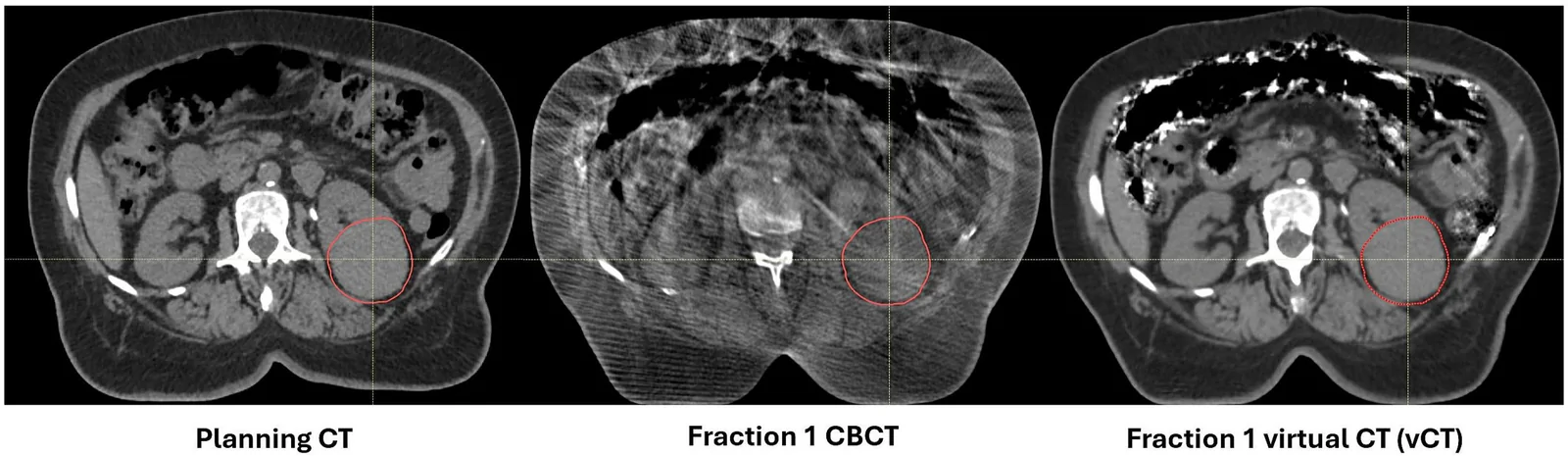
Sample virtual CT generation. The HU values were mapped from the treatment planning CT scan to the anatomy in the day-of-treatment CBCT scan, generating the virtual CT (vCT). The target (iCTV) is delineated in red. (For interpretation of the references to colour in this figure legend, the reader is referred to the web version of this article.)

CBCTs were deformably registered to the planning CT. Virtual CT sets were generated using a built-in workflow in the TPS, in which CT numbers from the simulation scan were mapped to the day-of-treatment CBCT (Fig. 2). The resultant virtual CT sets thus used anatomical information from the daily CBCT, with CT numbers from the planning image set.

**Fig. 3:**
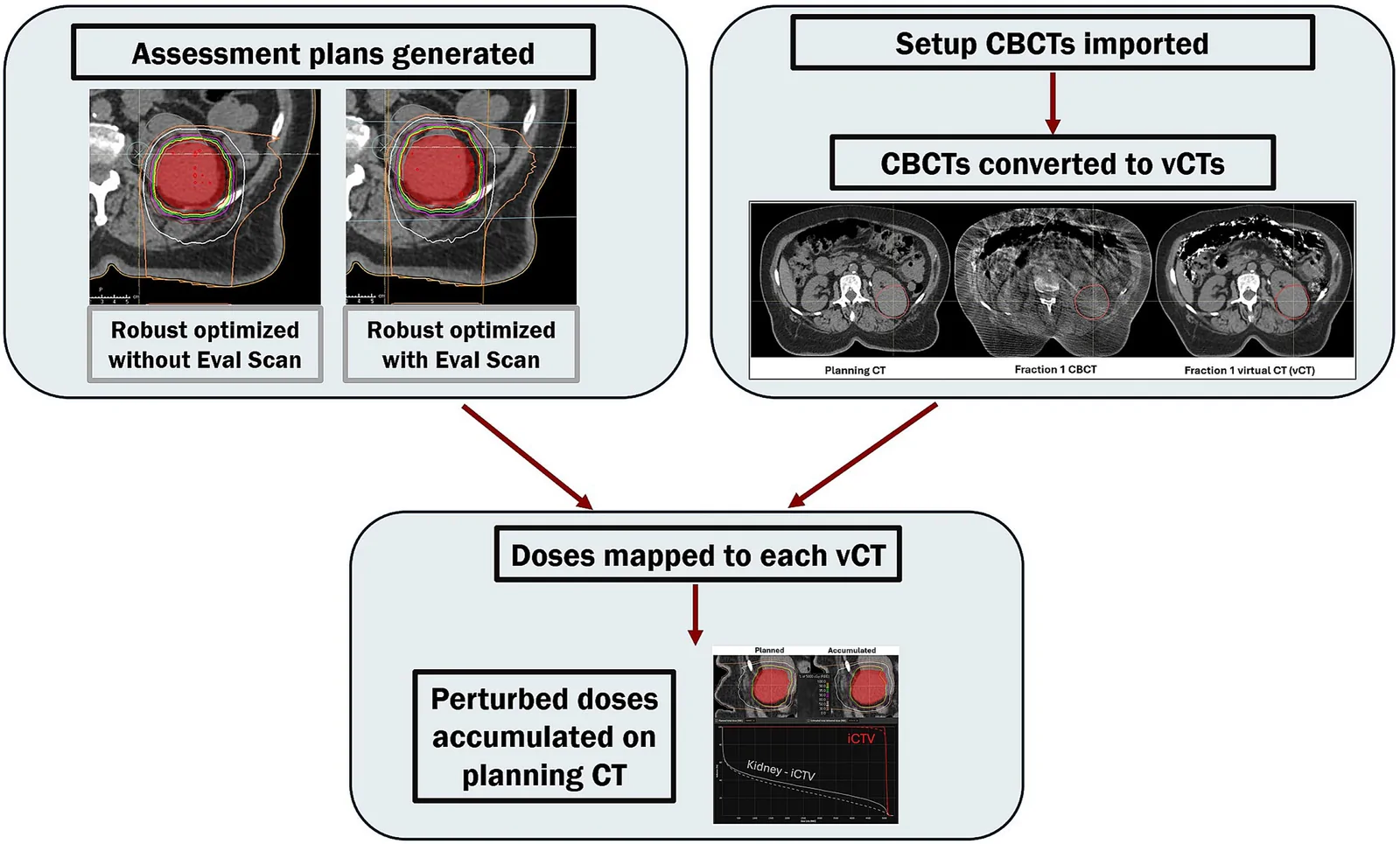
Study workflow diagram. Assessment plans were generated with and without the evaluation CT image set factored into robust optimization. In parallel, setup cone beam CT (CBCT) image sets were imported and converted to virtual CT (vCT) sets. Plan doses were mapped to each vCT, and accumulated on the planning CT for analysis.

The dose mapping and accumulation workflow is shown in Fig. 3. Fraction doses were calculated on each virtual CT. A final deformable registration was performed between each virtual CT and the planning CT for dose accumulation. Dose volume histogram (DVH) metrics were compared between the accumulated and nominal assessment plan dose. For targets, the dose to 95%, 98%, 99%, and 100% of the iCTV (D95, D98, D99, D100, respectively) were recorded. Additionally, the volume of ipsilateral nontumor kidney receiving at least 10, 20, 30, and 50 Gy (RBE) (V10, V20, V30, and V50) were recorded for comparison.

The distance between the iCTV centroid and the skin surface along each beam direction was measured on each virtual CT and recorded to characterize daily changes in depth.

### Statistics

Descriptive statistics were reported for evaluation of central tendency and dispersion. Comparisons between assessment plans and accumulated dose metrics were performed using paired two-tailed *t*-tests. The effect size was reported as Cohen's d, interpreted as negligible (<0.20), small (0.20–0.49), moderate (0.50–0.79), or large (≥0.80). Pearson correlation was used to assess relationships between perturbations and patient BMI or target volume. Statistical significance was defined as *p* < 0.05. Data analysis was performed in STATA version 18.0 (StataCorp, College Station, TX, USA).

## Results

### Patient cohort

Fifteen patients were included in the study. CBCT images for 75 total fractions were converted to virtual CTs. Patient characteristics are summarized in Table 1.

**Table 1:** Summary of patient and tumor characteristics.

Variable		Value or number of patients
Age (*N* = 15 patients)		Median 70 years) (Range: 53–92 years)
Height		171.9 ± 8.4 cm
Weight		90.7 ± 19.7 kg
BMI		30.6 ± 6.1
iCTV		75.2 ± 54.9 cc
Healthy kidney vol. (kidney-iCTV)		142.1 ± 53.4 cc
Tumor histology	RCC, NOS	6
	pRCC	4
	ccRCC	3
	ccpRCC	1
	Urothelial Carcinoma	1
Treated kidney	Right	6
	Left	9
Tumor location (relative to renal hilum)	Medial	2
	Lateral	3
	Central (right-left)	10
	Inferior	4
	Superior	6
	Central (inferior-superior)	5
	Anterior	1
	Posterior	11
	Central (anterior-posterior)	3

Abbreviations: BMI = body mass index; iCTV = internal clinical target volume; RCC = renal cell carcinoma; NOS = not otherwise specified; pRCC = papillary renal cell carcinoma; ccRCC = clear cell renal cell carcinoma; ccpRCC = clear cell papillary renal cell carcinoma.

### Tumor centroid depth

Relative to the initial CT simulation scan, the change in the iCTV centroid's physical depth along the P/A and lateral beam directions averaged −0.9 ± 4.4 mm and 0.8 ± 4.6 mm, respectively. The average depth change over both beam directions was 0.0 ± 4.6 mm. The mean magnitude (absolute value) of depth changes was 3.2 ± 3.2 mm. No statistically significant differences were observed in depth changes between beam directions (*p* = 0.889, d = 0.02).

Physical depth changes ranged from 17.4 mm shallower to 12.7 mm deeper compared to the simulation scan anatomy. Of the 75 evaluated scans, physical depth changes along the posterior and lateral beam paths exceeded 5.0 mm in 17 (22.7%) and 12 (16.0%) fractions, respectively. Depth variations exceeded 1.0 cm in 7 fractions (9.3%), five of which had >1.0 cm changes along the posterior beam path. Of the 150 total beams, 74 (49.3%) had depth variations under 2.0 mm.

### Tumor dose perturbations

**Fig. 4:**
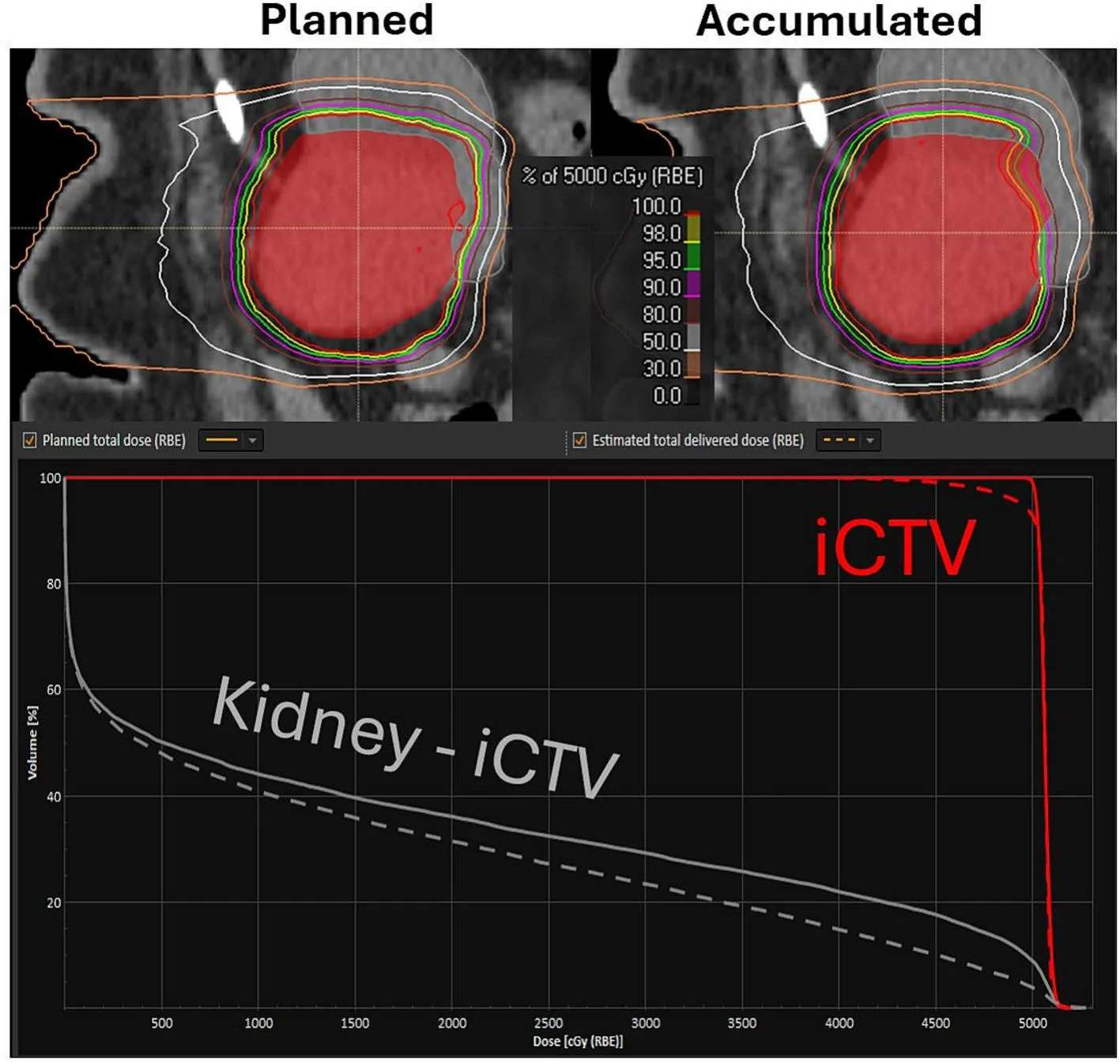
Planned and accumulated dose for the case with the third largest average tumor centroid depth variation. Top left: sagittal view of the planned dose distribution iCTV is denoted in the red colour wash. Top right: sagittal view of the accumulated dose distribution. Bottom: dose volume histogram (DVH) showing the planned (solid line) and accumulated (dotted line) curves for the iCTV and healthy kidney. (For interpretation of the references to colour in this figure legend, the reader is referred to the web version of this article.)

Statistically significant decreases in iCTV D95 (*p* < 0.01, d = 0.83), D98 (p < 0.01, d = 0.81), D99 (p < 0.01, d = 0.84), Dmin (p < 0.01, d = 1.01), and Dmax (p < 0.01, d = 1.51) were observed between the assessment plans and accumulated dose. The worst-case dose discrepancy at the prescription coverage level was D99 = 3787 cGy(RBE) (Table 2). The cases with the lowest accumulated D99 and Dmin had the third (4.7 ± 3.3 mm, Fig. 4) and fourth (4.1 ± 2.8 mm) highest average tumor centroid depth variation across fractions, respectively.

**Table 2:** Percentage changes in target dose metrics between assessment plans and accumulated dose for plans generated with and without the evaluation scan included in robust optimization. The lowest coverage dose and highest maximum dose are listed for each metric. *P*-value corresponds to the comparison in accumulated dose differences between optimizations.

Metric	No additional image sets		Evaluation scan included		*p*
Metric	% difference (SD)	Worst case dose [cGy(RBE)]	% difference (SD)	Worst case dose [cGy(RBE)]	
D95	−2.6 (3.2)	4553	−1.2 (1.4)	4828	0.04
D98	−5.1 (6.3)	4066	−2.9 (3.1)	4549	0.05
D99⁎	−6.7 (8.2)	3787	−4.4 (4.8)	4298	0.04
Dmin	−14.5 (14.5)	2641	−12.8 (12.2)	2854	0.15
Dmax	−0.5 (0.3)	5287 (max)	0.0 (0.9)	5493 (max)	0.05

⁎ = prescribed coverage level. Abbreviations: D95–99 = minimum dose covering 95–99% of the iCTV. Dmin = minimum dose covering 100% of the iCTV. Dmax = maximum point dose (D0.03 cc).

No statistically significant correlations between the patient's BMI and the change in iCTV D95 (*p* = 0.92), D98 (*p* = 0.91), or D99 (*p* = 0.87) between the nominal assessment plan and the accumulated dose were observed. The correlation between BMI and change in Dmin was weakly positive, though not statistically significant (r [13]=0.14, *p* = 0.63).

Correlations between the target volume and the change in iCTV D95 (*p* = 0.28), D98 (*p* = 0.14), D99 (*p* = 0.11), and Dmin (*p* = 0.09) were not statistically significant. The change in Dmax between accumulated and nominal assessment plan dose was positively correlated with target volume (r [13]=0.61, *p* = 0.02).

Significant differences between assessment plan and accumulated dose were observed in D95 (*p* < 0.01, d = 0.86), D98 (p < 0.01, d = 0.93), D99 (p < 0.01, d = 0.92), and Dmin (p < 0.01, d = 1.04) for the optimizations including the evaluation scan. Changes in the max dose were not significant (*p* = 0.98, d = 0.01).

Percentage differences between the assessment plan and accumulated dose were smaller for optimizations with the evaluation scan included for D95 (*p* = 0.04, d = 0.59), D98 (*p* = 0.05, d = 0.57), D99 (p = 0.04, d = 0.58), and Dmax (p = 0.05, d = 0.56). Differences in minimum coverage loss were not statistically significant between optimizations (*p* = 0.15, d = 0.39).

### Ipsilateral kidney dose perturbations

Assessment plans generated using the evaluation scan in robust optimization were associated with higher average healthy ipsilateral kidney V10 (*p* = 0.02, d = 0.66), V20 (p = 0.02, d = 0.68), V30 (*p* = 0.03, d = 0.64), V50 (p = 0.02, d = 0.68), and mean dose (p = 0.02, d = 0.68), though the V10-V50 differed by 1.0 cc or less on average. Kidney mean doses increased by 30.4 ± 44.7 cGy(RBE) when optimizing with the evaluation scan.

Accumulated dose changes in kidney V10, V20, and V30 were not statistically significant (*p* = 0.93, 0.41, 0.14, respectively), though volumes marginally reduced for assessment plans optimized without the evaluation scan (Table 3). On average, the kidney V50 dropped by 1.9 ± 2.0 cc (*p* < 0.01, d = 0.95). The accumulated mean kidney dose dropped by 50.3 ± 123.8 cGy(RBE) on average (*p* = 0.14), compared to the planned dose.

**Table 3:** Percentage changes in kidney (-iCTV) dose metrics between planned and accumulated dose for plans generated with and without the evaluation scan included in robust optimization. *P*-value corresponds to the comparison in accumulated dose differences between optimizations.

Metric	No additional image sets % difference (SD)	Evaluation scan included % difference (SD)	*p*
V10	−0.2 (6.0)	−0.1 (7.1)	0.69
V20	−1.8 (9.4)	−0.9 (10.4)	0.28
V30	−5.3 (13.5)	−4.1 (13.8)	0.25
V50	−37.1 (28.2)	−29.4 (30.5)	0.09
Mean	−3.2 (8.9)	−2.4 (9.9)	0.28

Abbreviations: V10-V50 = volume of kidney (-iCTV) receiving at least 10–50 Gy(RBE).

Similarly, when including the evaluation scan in robust optimization, changes in the V10, V20, and V30 with dose accumulation were not significant (p = 0.98, 0.77, and 0.37, respectively). The V50 decreased by 1.6 ± 2.4 cc (p = 0.03, d = 0.63). The accumulated mean dose decreased by 41.1 ± 147.5 cGy(RBE) on average, though the difference was not statistically significant (*p* = 0.30).

## Discussion

Variations in the skin surface perturb the delivered dose distribution. Though nearly half of beams (49.3%) had depth variations under 2.0 mm, discrepancies in physical depth of over 1.0 cm were observed in 9.3% of fractions, indicating a prevalence of large, but sporadic path length changes. Given the unpredictable nature of both the incidence and magnitude of variations, the skin surface should be monitored at each delivered fraction for all patients. Similar monitoring may also be indicated for treatments of other abdominal tumors which have significant loose tissue along the beam path.

The cases with the third and fourth highest average magnitude of depth variation were associated with the lowest D99 and Dmin after dose accumulation, indicating that repeated discrepancies were predictive of coverage loss. Therefore, the depth perturbations present a potential efficacy issue, as significant coverage loss may compromise local control outcomes. However, further studies correlating target coverage and clinical outcome are needed to fully characterize the effect.

The cohort was mainly comprised of patients with posterior kidney tumors. Since the irradiated volume of healthy kidney tended to decrease with dose accumulation, results demonstrate that skin surface variations tended to manifest in increased effective radiological path length. Though tumor centroid depth variations were centered around the planned physical depth (average 0.0 ± 4.6 mm), the use of a single sampling point may not have fully characterized the effect; however, changes in kidney dose metrics were small (Table 3), indicating minimal overall impact on organ dose.

In our treatment protocol, when significant anatomical variations are visualized on pre-treatment imaging, therapists reset and physically adjust the patient position to better match the reference anatomy before treatment delivery. In this assessment, the CBCTs used for virtual CT conversion were the initial scans taken before further adjustments to the patient position. Therefore, the assessment represents scenarios where no action was taken to address skin surface variations. Though prone positioning may mitigate posterior skin surface variations, patient tolerance and reproducibility issues in respiratory motion could adversely impact treatment quality. Prone positioning may be indicated for select cases in consideration of intended beam angles.

In treatment planning, the inclusion of the evaluation scan in robust optimization unanimously improved agreement between assessment plans and delivered dose (Table 2), demonstrating further value in repeat CT scans for evaluation of interfraction reproducibility. [24] However, evaluation scan anatomy may not be fully predictive of the variability seen on treatment, as demonstrated by instances of significant coverage loss (Table 2). Additionally, the inclusion of the evaluation scan in optimization increases the treated volume, potentially compromising organ sparing. Similarly, as some patients exhibited little skin surface variability, while other fractions had large discrepancies, unanimously increasing the range uncertainty in optimization may inappropriately increase the irradiated volume while inadequately protecting dose distributions against large variations.

Due to the unpredictability of depth variations, daily online adaptive radiotherapy (ART) may be an appropriate mitigation strategy. ART allows users to reoptimize plans based on day-of-treatment anatomy, [25], [26], [27] potentially enabling direct mitigation of skin surface variability. Furthermore, given that significant coverage loss was observed in our five-fraction regimens, the potential impact of daily variations may be greater for the 1- and 3-fraction regimens used in FASTRACK-II [9] due to reduced feathering effects. Online ART may be requisite for safe and maximally effective kidney SBPT. Further investigations of the feasibility and dosimetric benefit of online ART are planned in future work.

The study has several limitations which may impact interpretation of results. First, the assessment used nonclinical plans as the basis for dose accumulation. In practice, range uncertainty and additional 4DCT reconstructions are factored into robust optimization, along with the evaluation scan. These additional inputs may increase the overall robustness of clinical plans as compared to the tested optimizations. Second, the cohort was limited in size. Though representative of the cases encountered in our clinical practice, impacts on healthy kidney dose may vary depending on the tumor location. Furthermore, perturbations for anterior and lateral tumor locations may significantly impact dose to nearby bowel, meriting further consideration. Additionally, as most patients had high BMI (mean 30.6 ± 6.1), correlations between BMI and dose perturbation may be limited in interpretation for this cohort. An expanded study with differing tumor locations and patient characteristics is merited. Finally, uncertainties inherent to the virtual CT generation workflow may impact the calculated dose. [28], [29] The study cohort included patients with significant anatomical variation, and current CBCT image quality limitations may impact the fidelity of virtual CT generation. However, as algorithms and on-board imaging continue to improve, similar workflows as were used in this evaluation may be applied in online ART contexts.

## Conclusion

Daily skin surface variations can compromise SBPT treatment quality. Several mitigation strategies may be employed to ensure more consistent positioning, though the incidence and magnitude of skin surface variations were sporadic and unpredictable. Future studies are planned to assess the application of ART in kidney SBPT, which may enable safe treatment while maximizing the dosimetric benefits of PBT.

## Data sharing statement

The data that support the findings of this study are available from the corresponding author upon reasonable request.

## IRB statement

The study was conducted in accordance with the Declaration of Helsinki. The study was granted an exemption determination by the WCG IRB (00000533, #1-1785294-1). The requirement for informed consent was waived due to the study's retrospective nature.

## CRediT authorship contribution statement

**Yohan A. Walter:** Writing – original draft, Visualization, Validation, Software, Project administration, Methodology, Investigation, Formal analysis, Data curation, Conceptualization. **Olivia G. Moncrief:** Writing – review & editing, Validation, Software, Formal analysis, Data curation. **Chiachien J. Wang:** Writing – review & editing, Supervision, Resources, Investigation. **James C. Henry:** Writing – review & editing, Visualization, Software. **Mitchell Wolden:** Writing – review & editing, Visualization, Supervision. **Emily G. Warren:** Writing – review & editing, Visualization, Data curation. **Haley Clark:** Writing – review & editing, Investigation. **Amber Speir:** Writing – review & editing, Investigation. **Daniel B. Speir:** Writing – review & editing, Supervision, Resources. **Kelsi Hoffnung:** Writing – review & editing, Visualization, Investigation. **Bethany Broekhoven:** Writing – review & editing, Resources. **Philip F. Durham:** Writing – review & editing, Investigation. **Kaylee Kallam:** Writing – review & editing, Investigation. **Hsinshun T. Wu:** Writing – review & editing, Supervision, Software, Resources, Project administration, Methodology.

## Funding statement

No funding was received for this work.

## Declaration of competing interest

The authors declare the following financial interests/personal relationships which may be considered as potential competing interests: YAW, CJW, DBS, and HTW disclose active research agreement with CQ Medical (Avondale, PA, USA). YAW and DBS report receiving travel, speaker, consulting, and meeting registration funding from CQ medical. DBS reports receiving honoraria for consulting work with C-RAD AB (Stockholm, Sweden).
